# Sustained impact of subcutaneous immunotherapy among patients with allergic rhinitis who experienced treatment delay due to the COVID‐19 pandemic: A multicenter, two‐arm, real‐world study

**DOI:** 10.1002/clt2.12122

**Published:** 2022-03-01

**Authors:** Suizi Zhou, Yibin Liu, Jianrong Xue, Jun Tang, Qingqing Yu, Shenhong Qu, Shaojie Zhang, Binyu Mo, Jihui Li, Yinhong Liu, Yueying Yang, De‐Yun Wang, Qianhui Qiu

**Affiliations:** ^1^ Department of Otolaryngology‐Head and Neck Surgery, Guangdong Provincial People's Hospital Guangdong Academy of Medical Sciences Guangzhou China; ^2^ Department of Otolaryngology Zhujiang Hospital Southern Medical University Guangzhou China; ^3^ The Second School of Clinical Medicine Southern Medical University Guangzhou China; ^4^ Department of Otorhinolaryngology The Third People's Hospital of Changzhou Changzhou China; ^5^ Department of Otorhinolaryngology The First People's Hospital of Foshan Foshan China; ^6^ Department of Otorhinolaryngology People's Hospital of Guangxi Zhuang Autonomous Region Nanning China; ^7^ Department of Otorhinolaryngology Liuzhou People's Hospital Liuzhou China; ^8^ Department of Otolaryngology National University of Singapore, National University Health System Singapore Singapore

**Keywords:** COVID‐19, delayed therapy, depression, efficacy, subcutaneous immunotherapy (SCIT)

To the editor,

Subcutaneous immunotherapy (SCIT) is highly effective for seasonal pollinosis and perennial disease in patients with mite allergy.^1^ SCIT usually involves administering a gradually increasing dose of the specific allergens to allergic patients until the effective dose is reached then followed by administering the maintenance doses for 3 years or more.^2^ As the end of 2019 witnessed an outbreak of coronavirus disease 2019 (COVID‐19) in China, clinical, educational, research, and community responsibilities have been tremendously influenced in the whole world. Affected by the epidemic, many patients who were receiving SCIT were compelled to discontinue or postpone in the hospitals, which let us consider if any physical and mental impacts on patients with SCIT delayed during the COVID‐19 epidemic. In this research, we aim to follow up the physical and mental outcomes on SCIT delayed patients up to 1 year, which will enable us to develop novel strategies for SCIT management during the COVID‐19 epidemic.

Approval to conduct this study was obtained from the institutional review boards (2019‐KY‐106‐01). This study was a multicenter, two‐arm, real‐world study characterized by observational, prospective and nonrandomized. The study was performed in 643 allergic rhinitis (AR) patients with SCIT® [50% *dermatophagoides pteronyssinus* (Dp) and 50% *dermatophagoides farinae* (Df), Allergopharma Joachim Ganzer KG, Reinbek, Germany], who were IgE‐mediated sensitization to Dp or/and Df, between February 1 and May 31, 2020 (during the COVID‐19 outbreak in China) at the first visit (V0) and 319 patients were followed up at 1 year (V1). The clinical assessments included visual analogue scale (VAS),^3^ quality of life (QoL)^4^ and self‐rating depression scale (SDS)^5^ were collected by questionnaires (paper‐ or web‐based) for both V0 and V1. SCIT delayed is defined as an interval of more than 2 weeks in build up phase and more than 6 weeks in maintenance phase in this study, which match the suggestion by American Academy of Allergy, Asthma & Immunology (AAAAI).^6^ Statistical analyses were conducted with GraphPad Prism 7. Differences in clinical outcomes were compared using nonparametric Mann‐Whitney test. *p* value of < 0.05 was considered as statistically significant.

Of the 654 patients who were assessed for eligibility, 11 patients were excluded due to the lack of the record of the last injection date of SCIT in the questionnaire. Among them, 249 patients (38.72%) were received SCIT on schedule while 394 patients (61.28%) were postponed SCIT at V0. During V0 to V1, there were 161 patients (25.39%) completed SCIT (for more than 3 years) and 163 patients (25.35%) withdrew from SCIT. Thus, 105 patients on schedule (32.92%) and 214 patients delayed (67.08%) were followed up at V1. The time interval of the median value for SCIT delayed was 7 weeks, ranging from 1 to 30 weeks. The time for 83.6% of SCIT delayed patients was less than 10 weeks, 10–20 weeks for 14.5% patients, and only 1.9% patients were more than 20 weeks (Figure 1A).

**FIGURE 1:**
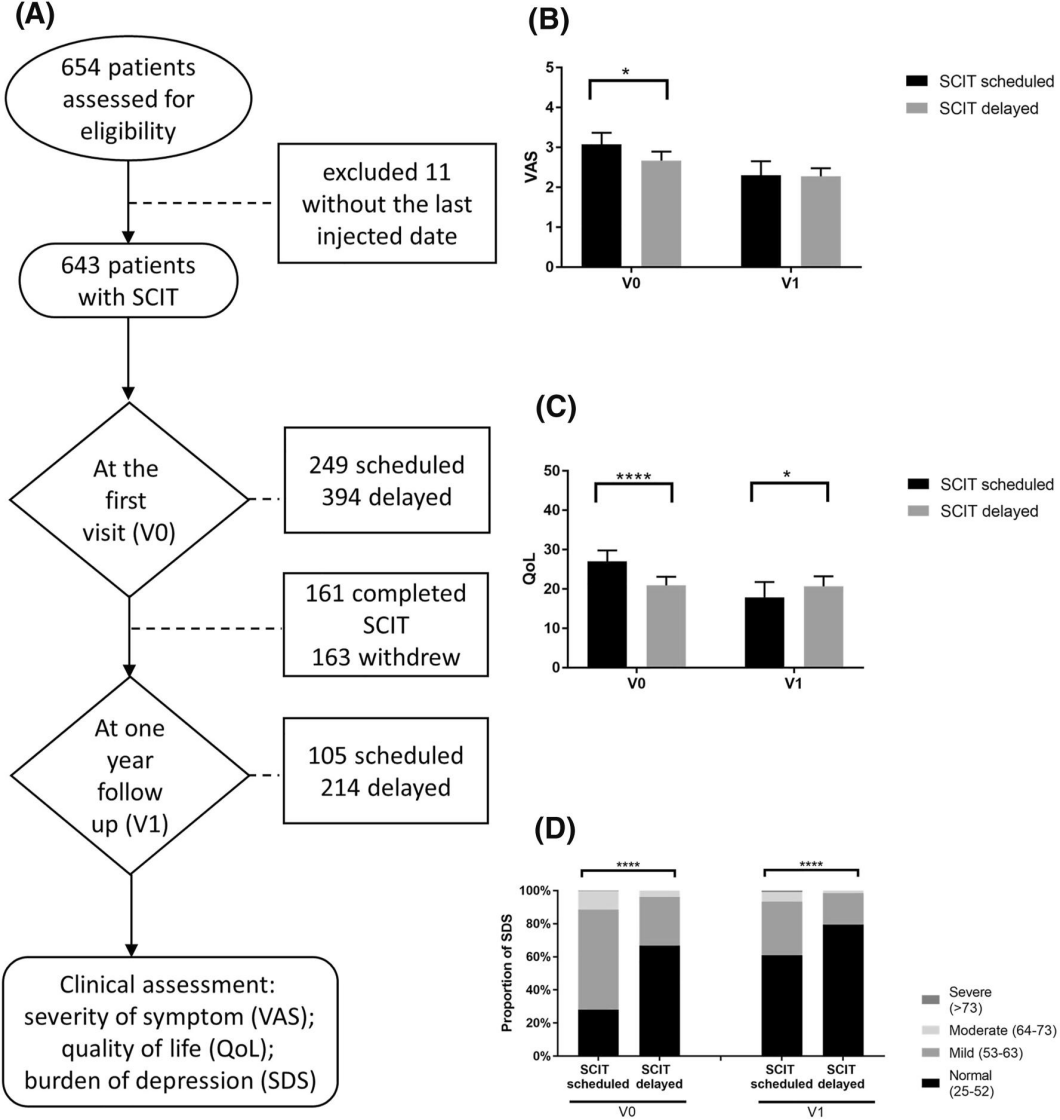
(A) Study flow chart. (B) Severity of symptom in patients with SCIT delayed compared to SCIT scheduled. (C) Quality of life in patients with SCIT delayed compared to SCIT scheduled. (D) Proportion of patients in depressed status with SCIT scheduled or delayed. Mean values with 95% CI are indicated by scale bar. ****p < 0.0001, ***p < 0.001, **p < 0.01, *p < 0.05. CI, confidence intervals; QoL, Quality of Life; SCIT, subcutaneous immunotherapy; SDS, self‐rating depression scale; V0, patients at the first visit; V1, patients at one year follow up; VAS, visual analogue scale

For subjective symptoms, the mean ± SD value of VAS was 2.67 ± 2.10 at V0 and 2.271 ± 1.53 at V1 for patients with SCIT delayed, whereas we found a higher score of 3.08 ± 2.13 at V0 (*P* = 0.0191) and 2.30 ± 1.82 at V1 for patients with SCIT scheduled (Figure 1B). For quality of life, the mean value with upper to lower 95% CI of QoL grade was 20.89 (18.73–23.05) and 26.97 (24.17–29.76) in patients with SCIT delayed and SCIT scheduled at V0, respectively (*P* < 0.0001). The remarkable upregulation in the grade of 20.71 (18.15–23.27) in patients with SCIT delayed in comparison to 17.85 (13.96–21.74) in patients with SCIT scheduled was seen at V1 (*P* = 0.0334), which showed a more life damage in SCIT delayed patients at V1 (Figure 1C). The score of clinical symptom and quality of life were still in the normal range above all.

The proportion of patients without depressed of SCIT scheduled and delayed were 28.11% and 66.86% at V0, and 60.95% and 79.52% at V1; mildly depressed patients were 60.33% and 29.33% at V0, 32.35% and 19.05% at V1; moderately depressed patients were 11.16% and 3.81% at V0, 5.80% and 1.43% at V1 (all *P* < 0.0001). Severely depressed patients were 0.40% and 0.90% at V0 and V1 respectively from SCIT scheduled, but none of them from SCIT delayed (Figure 1D).

First, this study confirms the long‐term efficacy of SCIT in AR patients even in patients with treatment delayed (less than 20 weeks), which is an important evidence that patients with delayed SCIT are encouraged to continue their SCIT during such a special time of COVID‐19 pandemic. Moreover, according to the depressed status of patients with SCIT scheduled and SCIT delayed, strengthening the importance of patient education and telemedicine, which need to be included as a part of medical practice especially in response to lockdown of hospital services in this critical period of COVID‐19 pandemic.

## CONFLICT OF INTEREST

The authors declare no potential conflicts of interest.
